# Effects of diet quality on the musculoskeletal system of the masticatory apparatus in *Mus musculus domesticus*

**DOI:** 10.1242/jeb.249735

**Published:** 2025-06-06

**Authors:** Levke Hansen, Daniela E. Winkler, Anja Guenther, Christine Böhmer

**Affiliations:** ^1^ Zoology and Functional Morphology of Vertebrates, Zoologisches Institut, Christian-Albrechts-Universität zu Kiel, Am Botanischen Garten 3-9, 24118 Kiel, Germany; ^2^ Max-Planck Institute for Evolutionary Biology, RG Behavioural Ecology of Individual Differences, 24306 Plön, Germany; ^3^ Department of Zoology and Animal Ecology, Hildesheim University, 31141 Hildesheim, Germany

**Keywords:** Feeding, Mandible, 3D geometric morphometrics, Quantitative anatomy, Rodents

## Abstract

Isolated environments such as islands can provide restricted dietary resources of varying quality. This has a major impact on evolution of island species, and can lead to rapid morphological adaptation, especially in small mammals. To understand the impact of diet quality on the masticatory apparatus in the model species *Mus musculus domesticus*, we quantitatively analysed the main masticatory muscles and the mandibular morphology in semi-natural populations kept on different diets for six generations. The investigation of individuals of the F5 generation raised on high quality (HQ) or standard quality (SQ) diets revealed significantly higher muscle mass and larger anatomical cross-sectional area (ACSA) of the m. masseter and the m. temporalis in mice raised on the SQ diet as compared with the HQ diet. A trend towards more robust (i.e. sturdy) mandible morphology in SQ mice as compared with HQ mice was evident. The investigation of individuals of two F6 generations that were fed on a diet different from that of the preceding generation revealed that the diet switch from HQ to SQ resulted in increased muscle size, whereas the diet switch from SQ to HQ lead to decreased muscle size as compared with the respective control group. The mandible displayed limited differences in morphology. These findings suggest that within six generations, diet quality could be a selection factor for morphological traits in the mandible that may become epigenetically fixed. However, additional studies such as DNA methylation and histone modification are necessary to unravel the role of the epigenome in this context.

## Teaser

**Summary:** In house mice kept under semi-natural conditions, diet quality influences the dimensions of the masticatory musculature rather quickly, whereas the morphology of the mandible appears less plastic over the same period of time.

## INTRODUCTION

Invasive species are one major component of global change in the Anthropocene (Murphy and Subedi, 2014; Daly et al., 2023). Invasion biology, the study of organisms that have been relocated outside their native geographical range as a result of human activities, currently offers several contrasting hypotheses aiming to explain the success or failure of species in novel habitats (e.g. Jeschke and Heger, 2018). Novel habitats often impose phenotype–environment changes, causing maladaptations, often leading to extinction (Bell, 2017). Successful individuals have been proposed to show so-called invasion syndromes: suites of traits that allow successful expansion and settlement (Chapple and Wong, 2016; Daly et al., 2023). To date, evidence for such invasion syndromes remain equivocal and are heavily biased towards the avian literature in most cases (Weis and Sol, 2016).

One well-known exception, for which ample studies exist also on terrestrial species with limited dispersal, is the colonisation of island habitats. Islands can be seen as natural laboratories that allow the observation of how evolutionary and spatial processes lead to biological diversity (reviewed by Losos and Ricklefs, 2009). The differentiation of species on islands was first studied by Charles Darwin (Darwin, 1845) and has since been the focus of numerous studies (e.g. Berry and Jakobson, 1974, 1975; Miller and Miller, 1995; Berry, 1968; Berry et al., 1978; Losos et al., 2006). The most obvious evolutionary trend observed is a change in body size, with small mammals exhibiting gigantism and large mammals tending to show dwarfism (Lomolino, 1985, 2005; Lomolino et al., 2012). This phenomenon has been termed the ‘island rule’ (Van Valen, 1973). Key factors contributing to the island rule were identified as (lack of) predation, food limitation, (lack of) interspecific competition, and selection for physiological efficiency (Heaney, 1978). Later, this concept was extended originally for rodent populations, which showed systematic differences in demography, reproduction, behaviour and morphology, with increased population density, and an infrequent production of few, large offspring, and termed island syndrome (Adler and Levins, 1994). Similar trends were also observed for large mammals, which displayed changes in life history, such as slower growth (Köhler et al., 2023), increased longevity and slower reproduction (Austad, 1993), reduction in size of brain and sensory organs as an adaptive strategy for more efficient use of energy (Köhler and Moyà-Solà, 2004), as well as a change to an energy saving locomotor behaviour in endemic mammals (Köhler and Moyà-Solà, 2003). Nowadays, the island syndrome has been found in diverse taxa, for example lizards (Novosolov et al., 2013), birds (Clegg and Owens, 2002; Ponti et al., 2023), insects (Leihy and Chown, 2020) and even plants (Biddick et al., 2019).

In fast reproducing small mammals such as rodents, morphological changes can occur relatively rapidly, within less than 100 generations, after introduction to an island habitat (Pergams and Ashley, 2001). Such changes not only encompass shifts in body size, but also a differentiation in mandible size between *Mus musculus* from different Faroe Islands (Berry et al, 1978) and sympatric *Apodemus* species from Japan (Renaud and Millien, 2001), a shift towards longer and wider snouts in rats and mice from different islands (Pergams and Ashley, 2001), changes in tooth size and shape in *M. m. domesticus* on the Canary Islands (Michaux et al., 2007), the Kerguelen Archipelago (Renaud et al., 2013, 2019) and the Orkney Archipelago (Ledevin et al., 2016), as well as an increase in cranial size in rats (Pergams et al., 2015). One of many possible reasons for changes in morphology is the difference in food availability and/or type of available food on secluded islands (Bozinovic and Rio, 1996; Renaud et al., 2010). Invasive rodent species often include a large proportion of invertebrates into their diet, and consequently consume more animal protein than plant protein as compared with their mainland counterparts (St Clair, 2011 and references within). Besides this larger availability of high-quality protein, the physical properties of the diet may also affect morphological changes in rodents: food consistency (hard or soft) has been shown to impact rates of bone formation and mineral apposition (Yamada and Kimmel, 1991) as well as the bone mineral density of the mandible in rats (Mavropoulos et al., 2005). Some evidence points in the direction that changes in muscles and in the morphology of the feeding apparatus can emerge even faster, even within one generation, potentially speeding up adjustment processes to novel or changing environments. For example, it was shown that a switch from hard to soft diet during ontogeny in rats results in higher stress on the bone structure (Mitchell et al., 2021). However, food consistency affects not only bone structure and density, but also the overall shape. Differences in food consistency caused changes in the mouse mandible morphology (Renaud et al., 2010), which also led to changes in mechanical advantage: mice raised on soft food diet showed a reduction in mechanical advantage compared with mice raised on a harder food diet, and the soft food group showed lower levels of integration between mandible regions (Anderson et al, 2014).

**Fig. 1.**
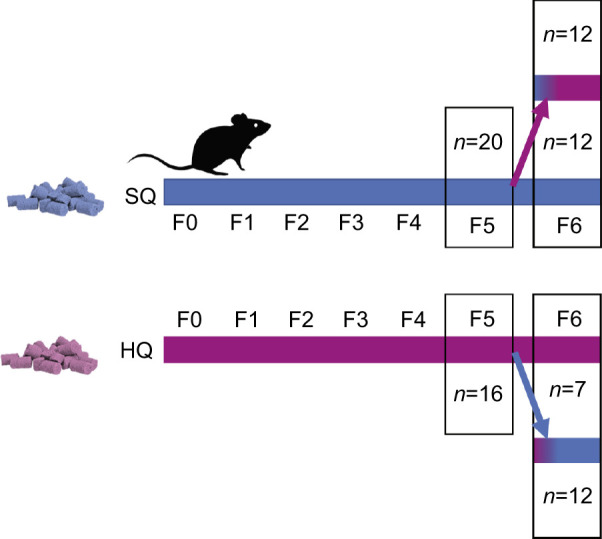
**Schematic representation of experimental design.** Mice were kept on either a standard quality (SQ) or high quality (HQ) diet for five generations. In the sixth generation, they were divided into four subgroups: control subgroups remained on their parents’ diet, while the switch subgroups were switched to the opposite diet.

As diet properties and energy content affect several of the traits involved in invasion/island syndromes such as life-history morphological and behavioural traits, we investigated how a difference in calorie content and hardness of food influences the musculoskeletal system of the masticatory apparatus in *Mus musculus domesticus*. Previous studies have shown that life history and behaviour already changed over the course of two generations when mice were either raised on a harder, standard quality (SQ) food (Altromin 1324) or a softer, higher quality (HW) food (Altromin 1414): mice on the HQ diet expressed higher fecundity rates (Prabh et al., 2023) and reduced risk-taking behaviour (Prabh et al., 2023; Porwal et al., 2023), as hypothesised to be a common phenomenon in small rodents invading novel habitats (Eccard et al., 2023). In comparison with SQ mice, HQ mice showed on average a better general body condition (Porwal et al., 2023). In addition to larger litters (5.5 pups per litter in HQ versus 3.2 pups per litter in SQ), offspring growth and survival rates were higher in HQ mice than in SQ mice (Prabh et al., 2023). Using descendants of house mice from the same setting, living in four replicates of semi-natural enclosures, we investigated different body and muscle parameters as well as the shape of the right mandibles of those mice raised on the SQ and HQ diet after five and six generations. Individuals of the F5 generation stayed on the same diet as their parents, whereas in the F6 only a group of individuals stayed on the same diet, while another group experienced the opposite diet from birth on (SQ to HQ, HQ to SQ; see Fig. 1 for an illustration of the experimental design). We focused on the two largest masticatory muscles, the musculus masseter and the m. temporalis, as the dominant jaw-closing muscles. Although the shape of a bone results from several processes, bone remodelling in response to muscle forces is thought to play a crucial role in shaping bone (Wolff, 1893). Through their attachment sites, the masticatory muscles can directly affect mandibular morphology over postnatal ontogeny (reviewed by Buvinic et al., 2020). Previously, Prabh et al. (2023) showed that SQ mice consumed more food in total as compared with HQ mice (per 24 h: SQ about 6.62 g and HQ 4.57 g; *F*=2.82, *P*=0.04). As the mice on the SQ diet need a higher food intake to reach their required calorie intake, we expected the muscles to be larger compared with the HQ group and thereby also undergoing a morphological change of the mandibles to a more robust (i.e. sturdy; possibly indicating greater strength) shape with more prominent muscle attachment areas. We also analysed whether a diet switch in the F6 results in a plastic change of mandible and muscle morphology as the development of the muscles also affects the shape of the mandibles (Sharir et al., 2011). Bone changes should follow changes in muscle and vice versa presumably because of the biomechanical link between them (e.g. reviewed by Buvinic et al., 2020). However, there is evidence that muscle and bone can be uncoupled (Turner, 2000).

## MATERIALS AND METHODS

### Animals and housing

*Mus musculus domesticus* Schwarz and Schwarz 1943 of the F5 generation were derived directly from semi-natural enclosures, from a study running for several generations. Although some animals were euthanised directly (all F5 samples here, *N*=36), others were moved to cage housing and used to establish breeding pairs after an initial habituation period to the new housing system. All F6 samples used here (*N*=43) represent the cage-born offspring of these breeding pairs. Upon euthanisation, all animals were frozen at −20°C until further processing.

Four semi-natural enclosures were originally founded by 20 unrelated males and females each and allowed to breed freely for five generations (for details, see Prabh et al., 2023). Semi-natural enclosures (17.5–19 m^2^ each) had wood-chip littering, offered 13 shelters for breeding as well as nine food and water stations each that offered food *ad libitum*. Temperature and daylight varied naturally but underfloor heating prevented temperatures from falling below 10°C to ensure animal welfare. Two enclosures received standard food (SQ; Altromin 1324). The founding animals as well as their parents had been raised on this type of food. The other two enclosures received a novel and high-quality food (HQ; Altromin 1414). The SQ diet contained 3227 kcal kg^−1^ metabolisable energy (24% from protein, 11% from fat and 65% from carbohydrates) whereas the HQ diet contained 3680 kcal kg^−1^ (28% from protein, 22% from fat and 50% from carbohydrates). The difference in food quality concerned the nutritional value, but also caused a slight difference in the hardness of the pellets.

During experiments, all animals born in the semi-natural enclosures were individually marked and parentage and generation were determined by microsatellite analyses. Genetic population structure, levels of heterozygosity and inbreeding in the enclosures were comparable to that of natural mice populations (Linnenbrink et al., 2018; Prabh et al., 2023). To obtain F5 individuals used here for morphometric analyses, we took adult females that had been reproductively active previously (i.e. healthy and in good body shape) pseudo-randomly, making sure that individuals were not closely related. Females were between 7 to 10 months old at the time of death.

Animals of the F5 generation removed for breeding were allowed to adjust to being housed in cages until we were sure that no female was pregnant or until females had successfully weaned their pups and animals showed no signs of being uncomfortable in cages. Cages were standard rodent type III Perspex cages (425×265×150 mm) filled with wood-chip bedding, a shelter made of egg carton, changing enrichment items as well as food and water *ad libitum*. Animals received natural daylight and temperatures varied between 16 and 22°C depending on season. Breeding pairs were set up by placing one female together with an unrelated male from the same food treatment. The male was removed when we could confirm pregnancy of the female (usually at ∼15–18 days after conception). From this time on, half of the females (SQ/HQ and HQ/SQ) received the respective different type of food. Thus, we established four treatments in F6 generation animals: SQ-control, HQ-control, SQ/HQ-switch and HQ/SQ-switch. At weaning with an age of 4 weeks, animals were separated into same-sex sibling pairs to prevent breeding. The F6 animals participated in a behavioural development study before being killed at the age of 4 months (±8 days). Thus, all F6 animals analysed here were of similar age and prior experiences.

We restricted the study to female animals of the F5 and F6 generations, as the diet switch was only introduced for F6 individuals, and hence for the previous generations no such comparative data would be available. By studying the F5 animals, we aimed to confirm whether an adaptation would already occur between two generations, i.e. by plasticity rather than selection pressures. The diet switch in the F6 was conducted to elucidate potential effects of developmental plasticity (Table 1).

**Table 1.** Overview of studied female individuals per diet group and generation

Diet group name	Diet in F0 to F4	Diet in F5	Diet in F6	Age (months)	*n*
F5 (female individuals kept in semi-natural environment)					
HQ	HQ	HQ	n.a.	7–10	16
SQ	SQ	SQ	n.a.	7–10	20
F6 (female individuals kept in cage)					
HQ-control	HQ	HQ	HQ	∼4	7
SQ-control	SQ	SQ	SQ	∼4	12
HQ/SQ-switch	HQ	HQ	SQ	∼4	12
SQ/HQ-switch	SQ	SQ	HQ	∼4	12

Two groups (HQ/SQ-switch and SQ/HQ-switch) experienced a switch from one diet to the other between two generations, i.e. during the last third of pregnancy. F, filial; HQ, high quality; *n*, number of analysed individuals; n.a., not analysed; SQ, standard quality.

### Ethics statement

All animals were handled and procedures were carried out under national and ARRIVE guidelines. Housing and breeding of mice were approved and are regularly controlled by the Veterinäramt Plön under permit 1401-144/PLÖ-004697. Procedures of population management under semi-natural conditions were approved under permit V244-12767/2019; behavioural experiments and killing of mice for organ withdrawal were approved under permit V244-31223/2019(62-5/19) by the former Ministerium für Energiewende, Landwirtschaftliche Räume und Umwelt, now called Ministerium für Landwirtschaft, ländliche Räume, Europa und Verbraucherschutz, Referat Tierschutz. Mice were killed by an overdose of CO_2_ before being stored at −20°C until further analyses.

### Body parameters

The intact dead bodies of the female mice were slowly thawed, and several body measurements were taken before dissection. All length measurements were acquired accurate to a millimetre with a ruler. Body length (BL) was measured from the tip of the snout to the base of the tail. Tail length (TL) was measured from the base to the tip of the tail. The length of the defleshed skull (SL) was measured from the most rostral to the most caudal point of the cranium. Body mass (BM) was obtained using an electronic scale (Kern) accurate to the nearest milligram.

### Quantification of the masticatory musculature

In mammals, there are four main muscles involved in mastication: m. masseter, m. temporalis, m. zygomaticus and m. pterygoideus. Because of the very small body size of mice, dissecting their masticatory muscles is very delicate work and has to be performed under the microscope. Damaging and losing muscle fibres when excising the muscle from the bone can negatively impact the muscle's mass and consequently may lead to uncertainties in subsequent calculations. In order to minimise such preparation bias and to keep the risk of damaging the bone as small as possible, we focused on the relatively largest masticatory muscles: m. masseter and m. temporalis (Baverstock et al., 2013). The m. masseter is specialised not only for gnawing as well as chewing, but also propalinal movement of the lower jaw, whereas the m. temporalis is mainly involved during incision (Becht, 1954). The m. masseter originates from the rostrum via a tendon attached to a small tubercle ventral of the infraorbital foramen, spanning the ventrolateral surface of the zygomatic arch, and inserts on both the ventromedial surface of the mandible (until the insertion of the m. ptergyoideus) and the ventrolateral surface of the mandible (masseteric fossa). The m. temporalis originates from the lateral surface of the cranium (covering the entire parietal bone) and inserts into the medial surface of the mandible (retromolar fossa and coronoid process).

After identification, the m. masseter was extracted as a whole and not divided into superficial and deep masseter. Similarly, the m. temporalis was not separated into the lateral and medial part, but instead treated as a m. temporalis complex. The muscles were rehydrated in water and pat dry on a paper towel to remove adhering water before measuring the muscle mass on an electronic scale (Kern) accurate to the nearest milligram. Afterwards, the length of the muscles was measured three times (accurate to the nearest half a millimetre) at the longest distance using a ruler and the mean of the three lengths was used for further analyses. The anatomical cross-sectional area (ACSA), which describes the area of a muscle in a plane perpendicular to its longitudinal axis (Sacks and Roy, 1982; Powell et al., 1984), was calculated as an indicator of muscle strength for the m. masseter and the m. temporalis using the following formula:
(1)

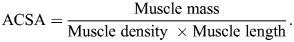


For this equation, 1.0597 g cm^−^³ was used as the standard value for muscle density (Méndez and Keys, 1960). The measurements for all muscle parameters were corrected to account for differences in size of the mice. The muscle mass and ACSA were corrected using the quotient of muscle mass (g) or ACSA (cm^2^), respectively, and the body mass. The muscle length (cm) was corrected by using the quotient of muscle length and skull length for further analyses.

### Quantification of the mandible shape

**Fig. 2.**
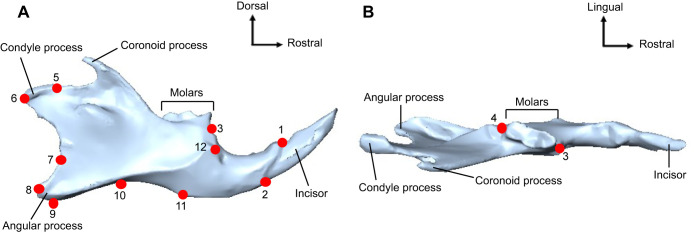
**Three-dimensional landmark set used in the geometric morphometric analysis of the mandible in *Mus musculus domesticus* (shown on specimen ID 972274200292804).** (A) Lateral view of right mandible with landmarks marked in red. (B) Dorsal view of right mandible for better representation of landmarks 3 and 4 marked in red. Refer to Table S1 for detailed landmark definitions.

To analyse differences in the shape of the mandibles from the different diet groups of the fifth and sixth generations, a three-dimensional (3D) landmark-based geometric morphometric analysis was performed on right hemimandibles. As we assumed bilateral symmetry in the craniomandibular morphology, left hemimandibles were not analysed. Digital surface models of the right hemimandibles were created using a 3D measurement system (Keyence VL-500, software VL-500 Series Application, version 2.2.13.97) with a display resolution of 0.1 μm, a precision of 2 μm and an accuracy of ±10 μm. The point clouds were converted into a mesh with approximately 80,000 vertices and 170,000 faces. Twelve homologous points (i.e. landmarks) were defined (Fig. 2) (modified after Pallares et al., 2014). These landmarks capture the morphology of the mandible in three dimensions and characterise relevant structures such as the incisor alveolus, the molar alveolus, the articular surface and the condyle, the angular process, the alveolar region, and the insertion region of the *M. masseter* (see Table S1 for detailed landmark definitions). The highly fragile coronoid process was not included in the landmark set as it was damaged for some of the specimens during the dissection.

The generated 3D surface models (*.ply) were loaded into the software IDAV Landmark (version 3.0, Wiley et al., 2005) and the landmarks were digitised on the surface of the models. Landmarking was performed by just one of us (L.H.). The 3D landmark coordinates obtained were exported, reformatted for import and aligned using a general Procrustes superimposition (Rohlf and Slice, 1990) in the software Morphologika^2^ (version 2.5).

### Statistical analyses

Data describing mouse body and muscle parameters were tested for normality using Shapiro–Wilk tests and inspected graphically with a normal probability plot in the software PAST (version 4.03, Hammer et al., 2001).

#### Distribution of body parameters per diet group

The distribution of body parameters per diet group was visualized via boxplots using the R package ggplot2 (version 3.3.0, Wickham, 2010) in R v. 4.2.1 (https://www.r-project.org/). As our data are non-parametric, Mann–Whitney pairwise *U*-tests were performed to reveal whether differences between the diet groups were statistically significant using the software PAST.

#### Masticatory musculature

Since phenotypic traits typically scale with overall body size, the muscle data were size-corrected by calculating the quotient between muscle parameter and body mass and skull length, respectively. The distribution of the size-corrected muscle parameters per diet group was visualized via boxplots. Since our data are non-parametric, it was tested for significant differences between the diet groups using Mann–Whitney pairwise *U*-tests using PAST. Muscle data are shown separately for left and right side in order to reveal any asymmetries or potential dissection bias.

#### Mandible shape

The superimposed 3D landmark coordinates were subjected to principal components analysis (PCA) in the software PAST (version 4.03, Hammer et al., 2001) to reveal the patterns of variation in mandibular morphology between the diet groups. Shape differences between the diet groups were investigated using a Procrustes ANOVA. The procedure is identical to permutational MANOVA as used in other fields (Anderson, 2001), but preferred for shape data. To do so, the procD.lm function from the R package ‘geomorph’ was applied (Adams and Otárola-Castillo, 2013). Additionally, boxplots were used to visualise the distribution of the individuals per diet group along PC1 and PC2, separately, and Mann–Whitney pairwise *U*-tests were used to test for significant differences between the diet groups using PAST. Shape changes along the PC axes were visualised in 3D showing individual PC scores in combination with wireframes using the R package ‘geomorph’ (Adams and Otárola-Castillo, 2013).

During Procrustes superimposition, all specimens are scaled to unit centroid size; i.e. the square root of the sum of the squared distances from each landmark to the centroid of each configuration (Zelditch et al., 2004). The natural logarithm (ln) of the centroid size was regressed against PC scores to reveal whether there are allometric shape differences in our sample. We also regressed body mass against PC scores to evaluate the relationship between these parameters.

#### Covariation between shape and muscle data

To test for covariation between mandible shape and muscle size, two-block partial least squares (two-block PLS) analyses were performed on the superimposed landmark coordinates of the mandible and the three tested muscle parameters, respectively. For the muscle parameters, the values for left and right side of the m. masseter and the m. temporalis were combined. The two-block PLS analyses were performed using the R package ‘geomorph’ (version 4.0.6, Adams and Otárola-Castillo, 2013). The F5 and F6 generations were analysed separately for all measured parameters as they cannot be compared directly owing to them being reared in different environments (semi-natural enclosures versus cages).

## RESULTS

### Body parameters

**Fig. 3.**
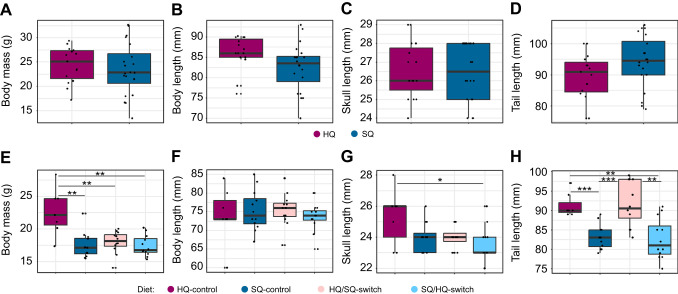
**Overview of distribution of body parameters for generations F5 and F6 sorted by diet group.** (A,E) Body mass, (B,F) body length, (C,G) skull length and (D,H) tail length. Each point represents one individual. Asterisks indicate significant differences as observed with the Mann–Whitney *U*-test (*P≤0.05, **P≤0.01, ***P≤0.001). Refer to Table 2 for an overview of the measured values.

In the F5 generation, mice kept on the HQ diet showed a trend towards longer bodies, but shorter tails compared with the mice that received the SQ diet (Fig. 3B,D, Table 2). No difference could be observed in the body mass and skull length between both diet groups (Fig. 3A,C, Table 2).

**Table 2.** Overview of measured body and muscle parameters shown as means*±*s.d.

	F5 generation		F6 generation			
Diet	HQ (*n*=16)	SQ (*n*=20)	HQ-control (*n*=7)	SQ-control (*n*=12)	HQ/SQ-switch (*n*=12)	SQ/HQ-switch (*n*=12)
BM (g)	23.57±4.14	25.55±5.28	22.64±3.32	17.49±1.89	17.99±1.64	17.41±1.52
BL (mm)	85.29±4.9	82.6±5.77	74.14±6.96	75.33±5.19	75.58±4.25	73.5±3.59
SL (mm)	26.53±1.43	26.4±1.46	25.29±1.67	24±0.91	24±0.71	23.5±1.12
TL (mm)	89.69±7.11	94.2±8.21	91.3±2.81	83.17±2.99	91.67±5.45	82.67±5.04
*M*_M_ (mg)	125.33±0.07	131.20±1.20	97.14±4	115±0.33	109.33±1.92	92.42±1.92
*M*_T_ (mg)	50.8±1.47	51.15±0.35	34.93±2.36	34.63±1.04	33.96±0.54	31.5±0.92
*L*_M_ (mm)	15.33±0.1	15.34±0.12	14.26±0.1	14.2±0.03	14.26±0.09	13.32±0.2
*L*_T_ (mm)	13.66±0.2	12.98±0.12	11.16±0.17	10.56±0.01	10.23±0.22	10.48±0.16
ACSA_M_ (mm^2^)	7.71±0.04	8.04±0.13	6.43±0.22	7.62±0.01	7.23±0.08	6.54±0.04
ACSA_T_ (mm^2^)	3.51±0.05	3.72±0.05	2.95±0.16	3.08±0.09	3.13±0.02	2.83±0.05

For all muscle parameters, the mean and s.d. were calculated for the left and right side combined. HQ, high quality; SQ, standard quality; BM, body mass; BL, body length; SL, skull length; TL, tail length; *M*_M_, mass of m. masseter; *M*_T_, mass of m. temporalis; *L*_M_, length of m. masseter; *L*_T_, length of m. temporalis; ACSA_M_, anatomical cross-sectional area of m. masseter; ACSA_T_, anatomical cross-sectional area of m. temporalis; *n*, number of analysed individuals.

In the F6 generation, the pairwise comparisons indicated that the body mass (Fig. 3E, Table 2) was significantly influenced by diet between HQ-control and SQ-control (*P*=0.004), HQ-control and HQ/SQ-switch (*P*=0.006), and HQ-control and SQ/HQ-switch (*P*=0.003). The body length (Fig. 3F, Table 2) showed no significant difference between diets. The skull length (Fig. 3G, Table 2) showed a significant difference between HQ-control and SQ/HQ-switch diets (*P*=0.04). Pairwise comparisons also indicated a significant influence of diet on the tail length (Fig. 3H, Table 2) in F6 between HQ-control and SQ-control (*P*=0.0006), HQ-control and SQ/HQ-switch (*P*=0.006), SQ-control and HQ/SQ-switch (*P*=0.0005) and HQ/SQ-switch and SQ/HQ-switch (*P*=0.002).

### Masticatory musculature

**Fig. 4.**
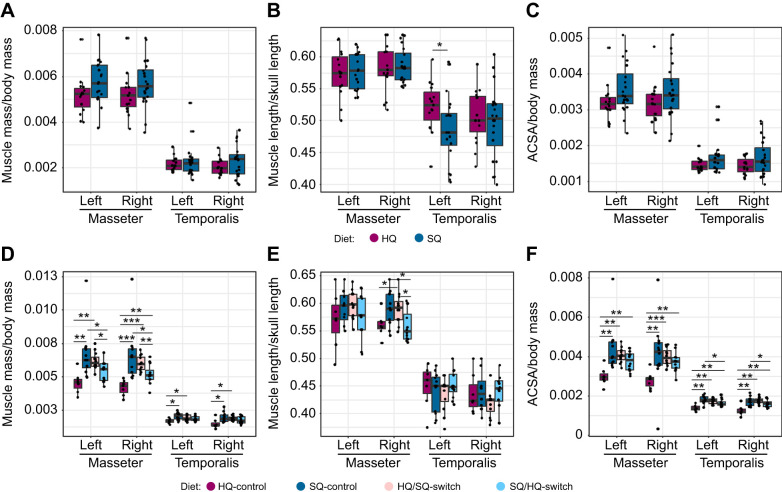
**Overview of muscle parameters for the m. masseter and m. temporalis of the left and right side of the head for generations F5 and F6 sorted by treatment group.** Each dot represents one individual. (A,D) Corrected muscle mass, (B,E) corrected muscle length and (C,F) corrected anatomical cross-sectional area (ACSA). Asterisks indicate significant differences as observed with the Mann–Whitney *U*-test (*P≤0.05, **P≤0.01, ***P≤0.001).

In the F5 generation, muscle mass and ACSA of left and right muscles were larger in the SQ group compared with the HQ group (Fig. 4A,C). The length of the m. masseter on both sides did not differ between diet groups (Fig. 4B). The m. temporalis were slightly shorter in the SQ diet group compared with the HQ diet (Fig. 4B) but showed no significant differences between both diet groups in mass or ACSA (Fig. 4A,C). In the F6 generation, muscle mass and ACSA of the m. masseter as well as the m. temporalis were significantly influenced by diet (Fig. 4C). The pairwise comparisons showed significant differences between HQ-control and SQ-control (left: *P*=0.002, right: *P*=0.0005), HQ-control and HQ/SQ-switch (left: *P*=0.003, right: *P*=0.0005), HQ-control and SQ/HQ-switch (right: *P*=0.006), SQ-control and SQ/HQ-switch (left: *P*=0.023, right: *P*=0.014), and HQ/SQ-switch and SQ/HQ-switch (left: *P*=0.019, right: *P*=0.007) for m. masseter mass and between HQ-control and SQ-control (left: *P*=0.016, right: *P*=0.016), and HQ-control and HQ/SQ-switch (left: *P*=0.038, right: *P*=0.013) for m. temporalis mass. Significant differences could also be observed for the ACSA (Fig. 4F) of the m. masseter between HQ-control and SQ-control (left: *P*=0.002, right: *P*=0.002), HQ-control and HQ/SQ-switch (left: *P*=0.001, right: *P*=0.0008), and HQ-control and SQ/HQ-switch (left: *P*=0.005, right: *P*=0.005), and for the ACSA of the m. temporalis between HQ-control and SQ-control (left: *P*=0.001, right: *P*=0.003), HQ-control and HQ/SQ-switch (left: *P*=0.002, right: *P*=0.003), HQ-control and SQ/HQ-switch (left: *P*=0.008, right: *P*=0.008), SQ-control and SQ/HQ-switch (left: *P*=0.04), and HQ/SQ-switch and SQ/HQ-switch (right: *P*=0.046. For muscle length (Fig. 4F), significant differences could only be observed for the right m. masseter between HQ-control and HQ/SQ-switch (*P*=0.038), SQ-control and SQ/HQ-switch (*P*=0.024), and HQ/SQ-switch and SQ/HQ-switch (*P*=0.024).

### Mandibular shape

**Fig. 5.**
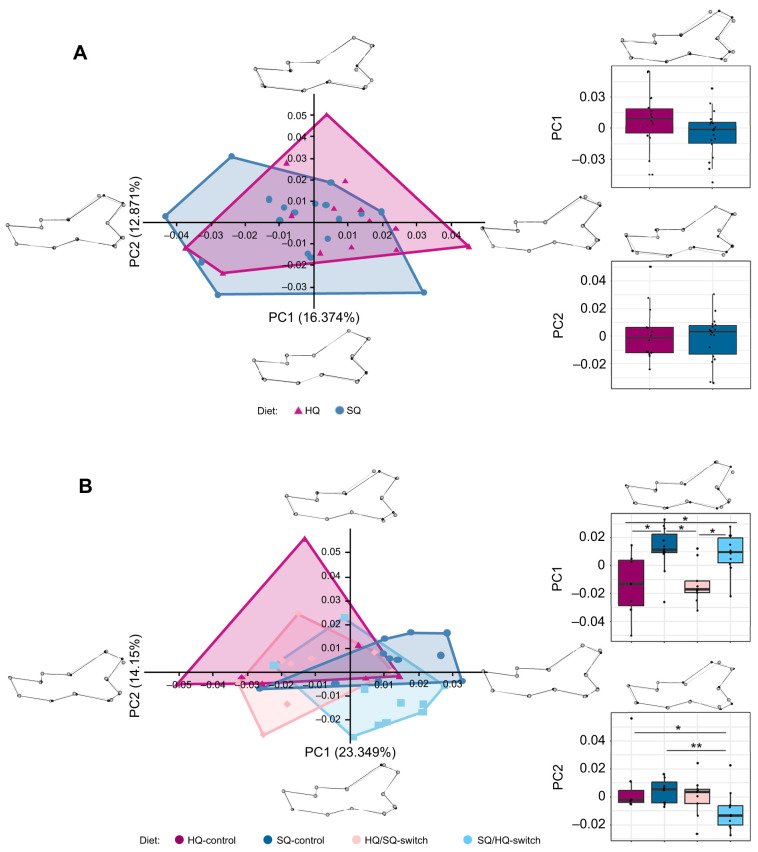
**Principal component (PC) analysis of right mandibles for generations F5 and F6.** (A) F5; (B) F6. Principle components 1 and 2 are shown. The diet groups are represented by differently coloured convex hulls: magenta for HQ, dark blue for SQ, pink for HQ/SQ-switch and light blue for SQ/HQ-switch. 3D shape changes along the PC axes are visualised using the extreme points for each axis in comparison to the mean shape. Boxplots show significant differences between diet groups for each principal component. Differences in mandible shape between minimal and maximal PC values for PC1 and PC2 are shown above the respective boxplots. Asterisks indicate significant differences as observed with the Mann–Whitney *U*-test (*P≤0.05, **P≤0.01, ***P≤0.001).

Considering all Procrustes superimposed landmark coordinates for the F5 generation, we observed no significant relationship between mandible shape and diet group (*F*=0.8568, *P*=0.649). Focusing on the first PCs, a trend appeared. For the F5 generation, PC1 and PC2 accounted for 29% of the total variation in mandible shape. Mandible shape of individuals kept on HQ and SQ largely overlapped; however, a trend was noticeable in SQ mice extending more into the negative *x*-values and HQ mice extending more into the positive *x*-values for PC1 (Fig. 5A). Negative values of the *x*-axis reflect a more robust mandible shape, especially the region of the condyle and angular process. Positive values of the *x*-axis represent a more gracile, elongated mandible shape. Along the *y*-axis, mandible shape mainly varied in the position of the most anterior concave point and the shape of the curve between the condyle and angular process: positive values are associated with the most concave point being closer to the angular process whereas negative values are associated with the most concave point being closer to the condyle. PC3 and PC4 accounted for 21% of the total variation in mandible shape (Fig. S1A). They revealed a similar pattern as PC1 and PC2, but there was more overlap between the diet groups. The following PCs each explained ≤7% of the total variation in mandible shape and are not considered here. The mandible shapes of HQ and SQ mice showed a large overlap, with SQ leaning more towards negative *x*-values, but no significant difference were observed between the diet groups in either PC3 or PC4.

The multivariate regression of ln centroid size against the PC scores revealed no significant allometry in shape differences except for PC7 (see Table S2). PC7 accounted for only 5.9% of the total observed variance in mandible shape and the coefficient of determination was rather low (*r*²=0.124, *P*<0.05). Likewise, the multivariate regression of body mass against the PC scores revealed no significant relationship (see Table S3).

Considering all Procrustes superimposed landmark coordinates for the F6 generation, we observed a significant relationship between mandible shape and diet group (*F*=2.0936, *P*=0.001). PC1 and PC2 accounted for 37.5% of the total observed variance in mandible shape. The diet groups displayed distinct differences between individuals kept on their parent generation's diet, and those switched to the opposite diet (Fig. 5B). Our results showed significant differences in PC1 between HQ-control and SQ-control (*P*=0.024), HQ-control and SQ/HQ-switch (*P*=0.045), SQ-control and HQ/SQ-switch (*P*=0.008), and HQ/SQ-switch and SQ/HQ (*P*=0.016), and in PC2 between HQ-control and SQ/HQ-switch (*P*=0.022), and SQ-control and SQ/HQ-switch (*P*=0.007). Here, HQ-control and HQ/SQ-switch mice extended more towards negative *x*-values in PC1, whereas SQ-control and SQ/HQ-switch mice extended more towards positive *x*-values. Shape differences in PC1 were influenced by the position of the condyle, whereas shape differences along PC2 were influenced by the length of the articular surface of the condyle. PC3 and PC4 accounted for 20% of the mandible shape variation (Fig. S1B). They revealed a similar pattern as PC1 and PC2, but there was more overlap between the diet groups. The other PCs explained ≤7% and are not considered here. The pairwise comparisons showed a significant difference between SQ-control and HQ/SQ-switch in PC4 (*P*=0.015), with SQ-control extending towards positive *y*-values whereas HQ/SQ-switch extends towards negative *y*-values, but besides that the four diet groups all clustered together.

The multivariate regression of ln centroid size against the PC scores revealed no significant allometry in shape differences except for PC1 (see Table S2). PC1 accounted for approximately 23.35% of the total observed variance in mandible shape and the coefficient of determination was intermediate (*r*²=0.519, *P*<0.0001). Likewise, the multivariate regression of body mass against the PC scores revealed no significant relationship except for PC1 (see Table S3). Shape differences along PC1 were slightly associated with body mass differences (*r*²=0.265, *P*<0.001).

### Covariation in the musculoskeletal system of the mandible

**Fig. 6.**
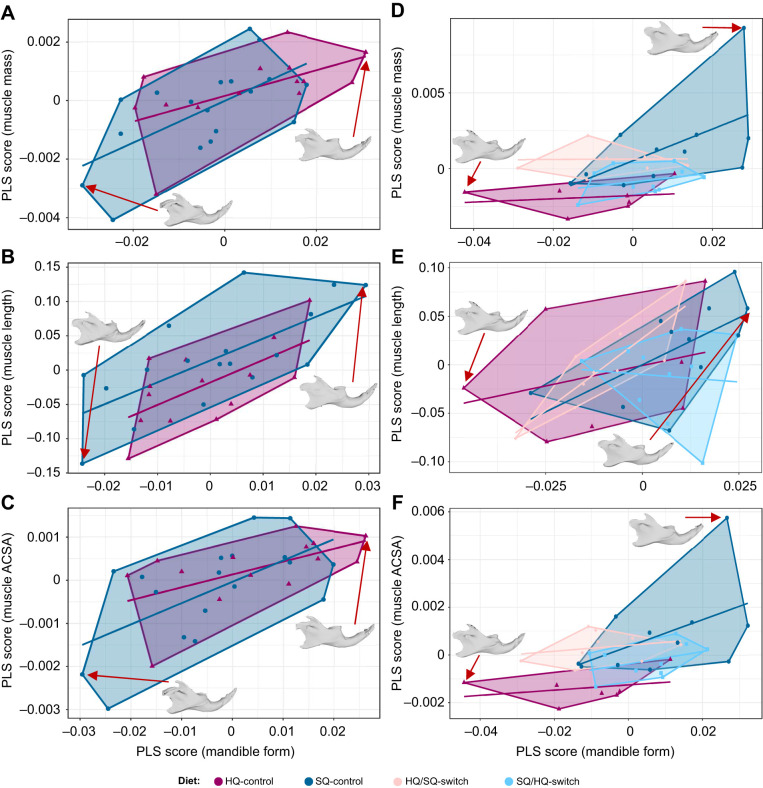
**Two-block partial least square (PLS) analysis of mandible shape to different muscle parameters in generations F5 and F6.** Correlations of mandible shape and (A,D) muscle mass, (B,E) muscle length and (C,F) and muscle ACSA for generations (A–C) F5 and (D–F) F6. The treatment groups are represented by differently coloured convex hulls: magenta for HQ, dark blue for SQ, pink for HQ/SQ-switch and light blue for SQ/HQ-switch. Correlations between the tested parameters are represented by lines. Red arrows indicate individuals that show the morphological differences in mandible shape between the minimal and maximal PLS scores in the shape of lateral views of 3D models of the specimen.

In the F5 generation, covariation between mandible shape and the three muscle parameters per diet group was relatively high, but not significant (Fig. 6A–C, Table 3). The strongest association was found for ACSA and mandible shape of HQ-control (rPLS=0.814, *P*=0.228). In the F6 generation, covariation between mandible shape and the three muscle parameters per diet group was also relatively high, but not significant, except for muscle length in the HQ/SQ-switch group (Fig. 6D–F, Table 3). The relationships differed between the diet groups (Fig. 6D–F). For example, individuals of HQ-control and HQ/SQ-switch share similar mandible shape, but they differ in ACSA (Fig. 6F). Likewise, individuals of SQ-control and SQ/HQ-switch largely share mandible shape and differ in ACSA (Fig. 6F).

**Table 3.** Overview of results of the statistical analyses for the partial least square (PLS) analysis (see Fig. 6) of mandible shapes of right hemimandibles to different muscle parameters

Muscle parameter	Diet	% Covariation to mandible shape	rPLS	*P*-value	rPLS_G_	*P*-value_G_
F5						
Mass	HQ	89.37	0.479	0.628	0.809	0.246
	SQ				0.642	0.932
Length	HQ	67.87	0.682	0.243	0.765	0.621
	SQ				0.8	0.257
ACSA	HQ	91.6	0.619	0.505	0.814	0.228
	SQ				0.627	0.949
F6						
Mass	HQ-control	96.95	0.45	0.821	0.81	0.504
	SQ-control				0.714	0.886
	HQ/SQ-switch				0.85	0.453
	SQ/HQ-switch				0.844	0.403
Length	HQ-control	66.34	0.438	0.943	0.787	0.633
	SQ-control				0.793	0.704
	HQ/SQ-switch				0.952	0.042*
	SQ/HQ-switch				0.896	0.182
ACSA	HQ-control	96.79	0.473	0.715	0.807	0.458
	SQ-control				0.688	0.941
	HQ/SQ-switch				0.861	0.375
	SQ/HQ-switch				0.837	0.442

The covariation, rPLS and *P*-value were calculated for all groups combined. rPLS_G_ and *P*-value_G_ were calculated for each diet group individually. *P<0.05.

## DISCUSSION

Our investigation of the body parameters, masticatory musculature and mandibular shape of *M. m. domesticus*, raised for several generations on either SQ or HQ diet, enabled us to identify phenotypic differences between the diet groups.

### Impact of diet quality on the masticatory apparatus

Animals obtain energy and nutrients from food and rely on their masticatory apparatus to acquire and process it. Through time, several mechanical solutions evolved depending on the feeding strategy and morphology of the masticatory apparatus, which can be linked to function (i.e. diet) (Bels and Wishaw, 2019; Schwenk, 2000). Yet, in mammals in particular, dietary flexibility also appears to be one of the main reasons for their success in exploiting different ecological niches and for their ability to undergo niche shifts in response to changing environmental conditions (e.g. Terry et al., 2017). House mice that arrived on Gough Island in the South Atlantic Ocean in the early 19th century adopted a predatory diet feeding on birds, causing wounds (Cuthbert et al., 2016; Parmenter et al., 2020) and even causing deaths of some birds (Connan et al., 2024). Morphological analyses revealed that Gough Island mice improved feeding performance by increasing the incisor biting leverage (Parmenter et al., 2020). In addition to availability, diet quality also affects the efficiency of energy intake, which is critical to the survival and reproductive success of animals (Townsend and Calow, 1981; Zhao and Wang, 2007). In our study, the musculoskeletal system of the masticatory apparatus in F5 mice was directly affected by differences in food quality. Even though the observed differences between diet groups were not significant, a trend was well visible. As the calorie content in the SQ diet is distinctly lower compared with the HQ diet, mice on the SQ diet need to consume larger quantities of food (on average 6.62 g per 24 h) as compared with mice on the HQ diet (on average 4.57 g per 24 h) (Prabh et al., 2023). This larger amount of food consumed supposedly increased the total time spent feeding, and thus the amount of chewing and gnawing. This is consistent with the observation of larger m. masseter mass and ACSA in mice on the SQ diet (Fig. 4A,C). Muscle mass and ACSA are proxies for the force-producing capacity of a muscle. Previous studies that have measured the forces developed by the m. masseter and diet-related changes *in situ* showed that muscle tension and activity were significantly lower in rats fed a soft diet than in those fed a harder diet (Kiliaridis and Shyu, 1988; Liu et al., 1998). The associated differences in m. masseter loading on the mandible affects bone remodelling, resulting in shape changes (Mavropoulos et al., 2004a,b; Abed et al., 2007; refer also to Buvinic et al., 2020 for a review on the concept of muscle–bone crosstalk in the masticatory system). We also observed differences in mandible shape between HQ and SQ mice in the F5 generation (Fig. 5A) that correspond to the lateral insertion of the m. masseter (i.e. ventrolateral surface of the mandible) amongst others. This part of the mandible is relatively larger in SQ mice, likely linked to the larger muscle mass and ACSA. This is in accordance with a previous study by Renaud et al. (2010) that found that the mandibular shape change in response to food consistency is rather localised around the molar zone and the insertion of the m. masseter in mice. Likewise, Bresin and Kiliaridis (2002) showed that reduced muscle capacity (owing to a soft diet) resulted in less bone apposition on the lower border of the mandible. Furthermore, our results reveal that the morphological differences covary with the observed differences in muscle mass and ACSA (Fig. 6A,B). Animal size (i.e. ln centroid size of the mandibular shape and body mass) does not account for the differences in morphology in the F5 generation.

### Rapid changes driven by diet switch

Radical changes in resource availability in isolated environments such as islands may drive rapid changes in phenotypic traits (e.g. Cucchi et al., 2014; Sagonas et al., 2014; Renaud et al. 2024). The results from our analyses of the F6 generation confirm this observation for the masticatory musculature. Similar to the results of the F5 generation, we found heavier and stronger m. masseter in SQ-control mice as compared with HQ-control mice of the F6 generation (kept in cages) (Fig. 4D,F). The general pattern of mandibular shape differences between the diet groups corresponds to that of the F5 generation (Fig. 5). The switch diet groups in the F6 generation showed significant differences from the control diet groups. Both the m. masseter and the m. temporalis of the switch diet groups tended to be more similar in mass and ACSA to mice receiving the food that the animals experienced after the food switch (i.e. HQ/SQ-switch was more similar to mice on SQ-control, and SQ/HQ-switch to mice on HQ-control diet rather than showing a ‘priming’ effect of food received in previous generations) (Fig. 4D,F). This supports the observation that muscle mass and ACSA are plastic and can change from one generation to the next in correspondence to environmental changes. However, contrary to muscle dimensions, the mandible shape of the switch diet groups was more similar to that of the parental generation (i.e. showing some lasting effects from previous generations; Fig. 5B). Our findings indicate that a diet switch leads to changes in muscle dimensions rather quickly by developmental plasticity, but may not affect the mandibular shape to the same extent or within the same time scale. Bone remodelling may take more time, either within the lifetime or may even take longer than just one generation (i.e. by transgenerational effects owing to alterations of the epigenome). Ödman et al. (2008) detected significant shape differences in the mandible between rats that were fed a soft diet and rats that were fed a hard diet for 7 months. However, the animals that switched in diet from soft to hard for a period of 6 weeks differed only marginally from the individuals that continued with a soft diet (Ödman et al., 2008). The authors assumed a longer rehabilitation period because they still observed a ‘catch-up’ tendency in mandibular shape change. The formation of adult morphology of the mandible is known to also be driven by factors such as epigenetic effects (Herring, 1993, 2011). It may be possible that the mandibular shape has been fixed in the epigenome of the previous generation and, thus, constrains the plasticity in the bone. However, only studies investigating, for example, DNA methylation and histone modification will allow us to unravel the role of the epigenome in this context (reviewed by Skvortsova et al., 2018). For instance, a study on cichlid fish from Lake Malawi assessed the role of an epigenetic mark in producing divergent phenotypes (DeLorenzo and Powder, 2023). It has been shown that inhibiting histone deacetylation using a drug (trichostatin A) affects the development of facial structures in cichlid fish from Lake Malawi (DeLorenzo and Powder, 2023). Additionally, it would be interesting to track the ontogenetic trajectory of the changes in masticatory musculature and mandibular morphology. Boell and Tautz (2011) found that shape differences between inbred strains already manifest early in ontogeny, and that later in life diet may only modulate already existing differences, instead of initiating them. Hence, the consistent mandibular shape observed in diet-switch individuals and their predecessors might indicate an inherited trait that is not marginally affected by dietary changes, whereas muscle morphology adapts plastically to the new diet quality. In F6, animal size (i.e. HQ mice and HQ/SQ mice are generally larger and heavier than SQ mice and SQ/HQ mice) had an influence on the morphology of the mandible (Fig. S2B). Yet, allometry does not explain all shape variation.

### Comparison between F5 and F6

Although the morphological analysis revealed the same differences in mandibular shape between SQ and HQ for the F5 and F6 generations (Fig. 5), the shapes were only significantly different in F6. In F5, there was more overlap between the two diet groups. Our landmark set is limited to capturing key features of mandibular morphology. Possibly, an extended landmark set may yield a clearer picture that reveals finer morphological differences.

In the F6 mice, the muscle parameters of HQ-control and SQ-control displayed the same trend as observed for the F5 generation. Interestingly, the HQ/SQ-switch group was similar in muscle parameters to the SQ-control, whereas the muscle parameters of the SQ/HQ-switch group revealed a trend towards decreased values but still resembled that of the SQ-control (Fig. 4). This indicates that the increase in muscle strength (approximated by muscle mass and muscle ACSA) happened quicker than the decrease.

In relation to body parameters, we observed different patterns in the two generations (Fig. 3): the HQ mice had shorter tails compared with SQ mice in the F5 generation, but in generation F6 we observed the opposite effect. Also, mice in the HQ-control diet group in the F6 generation were significantly bigger in body mass compared with the other diet groups, but we did not observe such effect in the F5 generation. Under semi-natural conditions, mice on the HQ diet were observed to start reproducing at the age of 3 months, whereas for mice on the SQ diet, the onset of reproduction was a couple of months later (Prabh et al., 2023). The cage-kept individuals of the F6 generation were not allowed to reproduce, hence HQ-control mice only invested energy in body mass gain and growth, whereas HQ mice in semi-natural environments had a larger energy expenditure for reproduction. However, Prabh et al. (2023) also noticed that life history strategies and risk-taking behaviours of the preceding generations of mice to our study differed between individuals kept on HQ and SQ diets. The correlation between life history traits and behaviour from the F1 generation was visible onwards in each subsequent generation and with increasing magnitude. A high-quality diet also led to male mice being preferred in mate choice compared with standard-quality diet mice (Porwal et al., 2023), indicating that differences in food quality influence different aspects of behaviour and life history traits. It can therefore be assumed that the development of the musculoskeletal system may also have been affected within the same time.

Overall, muscle mass and ACSA appear to underlie adaptive plasticity and change in correspondence to environmental changes, whereas mandible shape variations appear to be affected by remodelling through changes in muscle sizes as well as developmental plasticity. It is important to note that both the SQ and HQ diets were artificial pelleted feeds, and that therefore masticatory movements in our mice may differ from those of wild populations. However, previous studies have shown that pellet-fed mice were very similar in mandible shape to wild-captured mice (Boell and Tautz, 2011; Pallares et al., 2015). Therefore, our experimental results can be seen as a good analogue to possible effects observed in the wild.

### Conclusions

Our study revealed differences in the strength of the m. masseter (approximated by muscle mass and ACSA) in relation to dietary quality and associated changes in mandibular shape in experimental populations of *M. m. domesticus*. Studies investigating island syndromes have shown rapid morphological and behavioural changes occurring within in less than 100 years (Berry, 1968; Renaud et al., 2018; Li et al., 2021). Our data, combining semi-natural observations and controlled experiments investigating plasticity from one generation to the next, indicate that such phenotypic adjustments may happen even faster, within very few generations. The potential for fast, likely plastic, adaptation might explain the success of rodents in colonisation of new habitats. Diet as an environmental factor can influence phenotypes plastically, for example through epigenetic effects. Our results from the diet switch groups may hint at an epigenetic fixation of mandible shape remaining for one generation after experiencing a change of food.

Because the components of the masticatory system are highly coordinated and minimal deregulation in one of them can cause substantial alterations in the entire system, the present work also raised several questions to be investigated in the future. For example, do the observed differences in the masticatory musculature correlate with differences in mandibular bone mineral density? For instance, it has been shown that reduced masticatory muscle strength (as a result of soft diet) negatively affected alveolar bone microarchitecture in rats (Mavropoulos et al., 2004a,b). The lower muscle mass and ACSA in our HQ mice can potentially lead to resorption of the alveolar bone, which in turn negatively affects the dentition and can result in pathologies.

## Supplementary Material

10.1242/jexbio.249735_sup1 Supplementary information
