# Supplementary material for: Effects of diet quality on the musculoskeletal system of the masticatory apparatus in Mus musculus domesticus
Source: J Exp Biol. 2025 Jun 6;228(11):jeb249735. doi: 10.1242/jeb.249735 (PMC12188243; doi:10.1242/jeb.249735)
Supplement: Supplementary information [file jexbio-228-249735-s1.pdf]

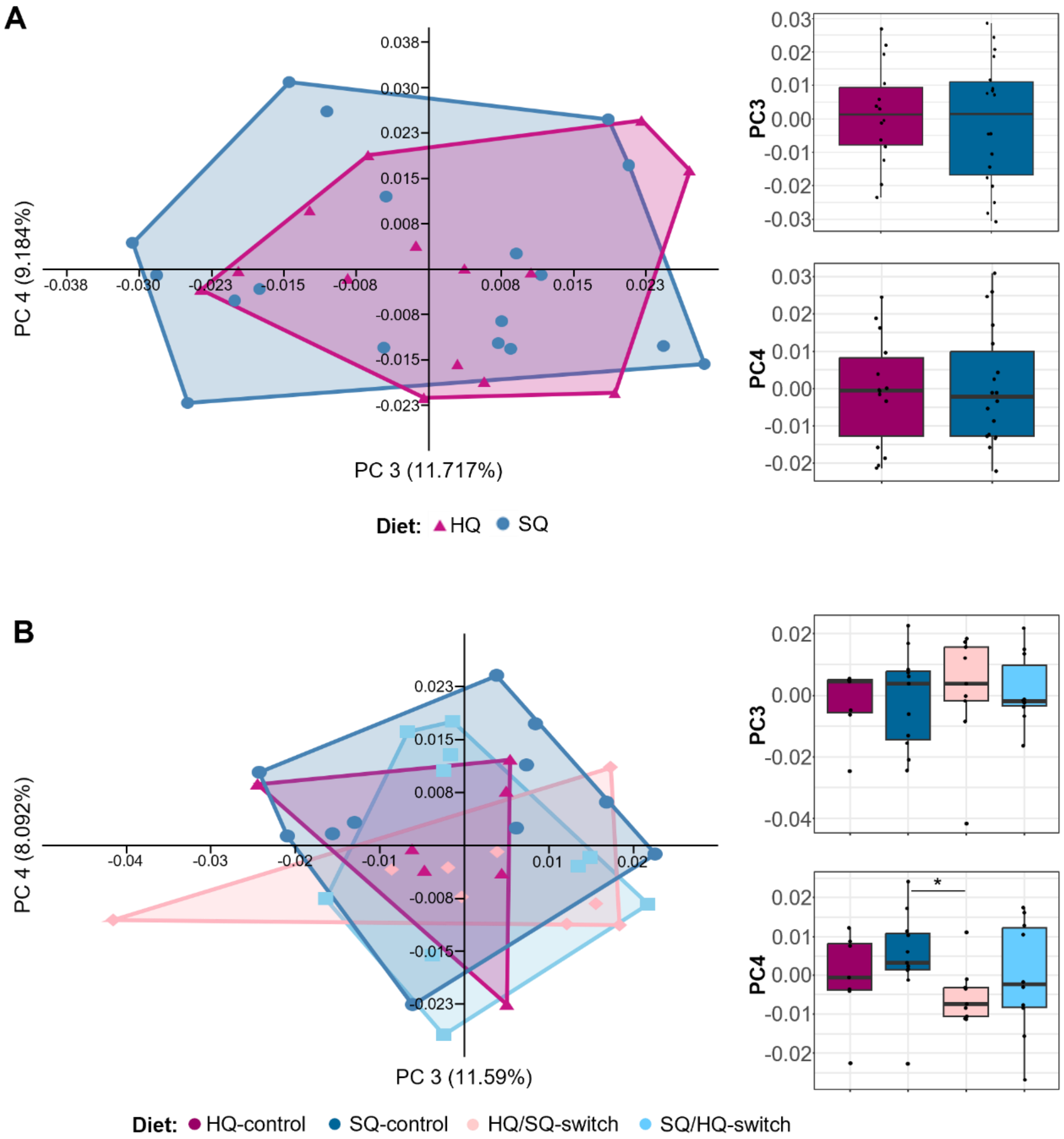

Fig. S1. Principal component analysis of right mandibles for generation A) F5 and B) F6. Principle components 3 and 4 are shown. The treatment groups are represented by differently coloured convex hulls: magenta for HQ, dark blue for SQ, pink for HQ switched to SQ and light blue for SQ switched to HQ. Form variations of extreme points for each axis are shown. Additionally, boxplots show significant differences between diet groups for each principal component. Asterisks indicate significant differences as observed with the Mann-Whitney U test. *P ≤ 0.05 and P > 0.01, **P ≤ 0.01 and P > 0.001, ***P ≤ 0.001.

Journal of Experimental Biology • Supplementary information

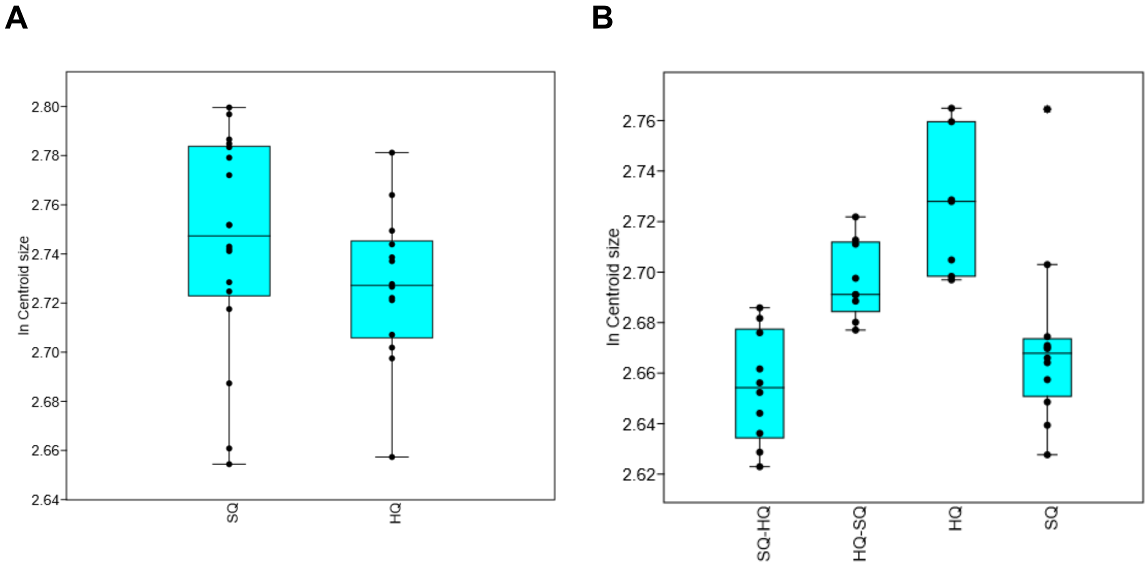

Fig. S2. Boxplots of ln Centroid size between diet groups for F5 and F6.

Journal of Experimental Biology • Supplementary information

Table S1. Definition of landmarks used in this study.

No.	Description
1	Most superior point of incisor alveolus
2	Most inferior point of incisor alveolus
3	Most anterior point of first molar alveolus
4	Most posterior point of third molar alveolus
5	Most anterior point of articular surface of the condyle
6	Most posterior tip of the condyle
7	Most anterior concave point between condyle and angular process
8	Most posterior tip of angular process
9	Most inferior point of angular process
10	Ascending inferior ramus dorsal most ventral point
11	Alveolar region most inferior point
12	Most anterior point of masseteric muscle insertion into the alveolar region

Table S2. Results of the multivariate regression of ln Centroid size against the first 10 PCs. Significant coefficients of determination (r^2) are highlighted in grey. Asterisk (*) indicates $p<0.05$.

Generation	Principal component	r^2	p-value
F5	~PC1	0.030	0.347
	~PC2	0.011	0.576
	~PC3	0.102	0.075
	~PC4	0.066	0.155
	~PC5	0.0001	0.963
	~PC6	0.063	0.165
	~PC7	0.124	0.048*
	~PC8	0.021	0.432
	~PC9	0.013	0.531
	~PC10	0.088	0.100
F6	~PC1	0.519	<0.0001*
	~PC2	0.002	0.770
	~PC3	0.008	0.605
	~PC4	0.003	0.762
	~PC5	0.037	0.244
	~PC6	0.0002	0.921
	~PC7	0.002	0.793
	~PC8	0.048	0.187
	~PC9	0.001	0.819
	~PC10	0.00003	0.972

Journal of Experimental Biology • Supplementary information

Table S3. Results of the multivariate regression of body mass against the first 10 PCs. Significant coefficients of determination (r^2) are highlighted in grey. Asterisk (*) indicates $p<0.05$.

Generation	Principal component	r^2	p-value
F5	~PC1	0.013	0.533
	~PC2	0.061	0.173
	~PC3	0.028	0.358
	~PC4	0.009	0.611
	~PC5	0.007	0.659
	~PC6	0.046	0.239
	~PC7	0.052	0.212
	~PC8	0.115	0.058
	~PC9	0.0004	0.906
	~PC10	0.161	0.023
F6	~PC1	0.265	0.001*
	~PC2	0.028	0.314
	~PC3	0.004	0.705
	~PC4	0.000	0.941
	~PC5	0.002	0.807
	~PC6	0.004	0.715
	~PC7	0.001	0.883
	~PC8	0.019	0.407
	~PC9	0.047	0.191
	~PC10	0.005	0.664

Journal of Experimental Biology • Supplementary information
